# Bivalent vaccines effectively protect mice against influenza A and respiratory syncytial viruses

**DOI:** 10.1080/22221751.2023.2192821

**Published:** 2023-05-09

**Authors:** Sathya N. Thulasi Raman, Adrian Zetner, Anwar M. Hashem, Devina Patel, Jianguo Wu, Caroline Gravel, Jun Gao, Wanyue Zhang, Annabelle Pfeifle, Levi Tamming, Karan Parikh, Jingxin Cao, Roger Tam, David Safronetz, Wangxue Chen, Michael J.W. Johnston, Lisheng Wang, Simon Sauve, Michael Rosu-Myles, Gary Van Domselaar, Xuguang Li

**Affiliations:** a Centre for Oncology and Regulatory Research, Biologic and Radiopharmaceutical Drugs Directorate, Health Products and Food Branch, Health Canada and WHO Collaborating Center for Standardization and Evaluation of Biologicals, Ottawa, Canada; b National Microbiology Laboratory, Public Health Agency of Canada, Winnipeg, Canada; c Vaccines and Immunotherapy Unit, King Fahd Medical Research Center, King Abdulaziz University, Jeddah, Saudi Arabia; d Department of Medical Microbiology and Parasitology, Faculty of Medicine, King Abdulaziz University, Jeddah, Saudi Arabia; e Human Health Therapeutics Research Center, National Research Council of Canada, Ottawa, Canada; f Department of Chemistry, Carleton University, Ottawa, Canada; g Centre for Vaccines Clinical Trials and Biostatistics, Biologic and Radiopharmaceutical Drugs Directorate, Health Products and Food Branch, Health Canada, Ottawa, Canada; h Department of Biochemistry, Microbiology and Immunology, Faculty of Medicine, University of Ottawa, Ottawa, Canada

**Keywords:** Adenovirus, influenza, RSV, pre-fusion stabilized F, HA-stem, RSV T-cell epitopes

## Abstract

Influenza and Respiratory Syncytial virus (RSV) infections together contribute significantly to the burden of acute lower respiratory tract infections. Despite the disease burden, no approved RSV vaccine is available. While approved vaccines are available for influenza, seasonal vaccination is required to maintain protection. In addition to both being respiratory viruses, they follow a common seasonality, which warrants the necessity for a concerted vaccination approach. Here, we designed bivalent vaccines by utilizing highly conserved sequences, targeting both influenza A and RSV, as either a chimeric antigen or individual antigens separated by a ribosome skipping sequence. These vaccines were found to be effective in protecting the animals from challenge by either virus, with mechanisms of protection being substantially interrogated in this communication.

## Introduction

Influenza and respiratory syncytial virus (RSV) are responsible for most viral respiratory tract infections in children [1,2]. Globally, the seasonality of both infections is known to overlap, especially in countries with a temperate climate [3,4]. Moreover, co-infections with the two viruses can result in generation of hybrid viral particles *in vitro* with altered antigenicity and tropism potentially impacting pathogenesis and disease outcome [5]. Considering the immense public health burden and the common seasonality of the two viral infections, it would be beneficial to synchronize public health interventions, such as vaccines, for both viruses. Consequently, vaccines targeting both viruses would greatly facilitate such synchronized public health measures.

The influenza Hemagglutinin (HA) protein comprises two subunits namely HA1 and HA2 [6], which together form the Head and Stem domains of the HA glycoprotein. While the globular head is mainly composed of the HA1 subunit, the stem domain spans both HA1 and HA2 subunits which flanks the N- and C-termini of HA1 [7]. As the most immunogenic and highly exposed region of HA, the head domain is subject to strong selective pressure, which results in high diversity among different strains. On the other hand, the HA stem domain is highly conserved, as it is occluded on the virion surface and subject to very little evolutionary pressure [8]. Many groups have tried to construct immunogenic “Headless” HA in pursuit of a universal influenza vaccine with varying degrees of success [9–14]. Thus, the stem domain is an attractive target for developing broadly protective and universal influenza vaccines [8,15]. Indeed, our lab has successfully tested the utility of the HA2 stem domain sequence in designing broadly protective vaccines against influenza viruses [16,17].

While vaccines against RSV are currently unavailable, many vaccine candidates are currently under clinical development and most target the Fusion (F) glycoprotein of RSV [18], due its conserved nature and necessity for virus entry [19]. It is well-established that the F protein exists as a metastable pre-fusion trimer on the virion membrane and most of the neutralizing antibodies in human sera are directed against the pre-fusion state of the trimeric protein [20,21]. Therefore, there has been a considerable interest in testing the pre-fusion stabilized RSV-F protein as a vaccine for RSV [22–26]. Besides neutralizing antibodies, CD4 and CD8 T cells also mediate and correlate with clearance of RSV in both mice and humans [27–30].

In this study, we designed and rescued adenovirus-based vaccine constructs encoding the gene sequences for the HA stem of influenza A H1 subtype and either the pre-fusion stabilized form of RSV-F or sequences of several known immunodominant and subdominant RSV T-cell epitopes. We tested the efficacy of these vaccine candidates and successfully demonstrate protection against both influenza A H1N1 and RSV virus challenge in BALB/c mice. To the best of our knowledge, this is the first study, where an adenovirus-vectored chimeric vaccine targeting both influenza and RSV has been shown to protect against both viruses, along with substantial investigation of the mechanism underlying the protection. Such proof-of-concept designs show promise as candidate vaccines for inducing broad-ranging protection against multiple pathogens and subtypes.

## Methods

### Cells and media

HEp-2 cells were cultured in Dulbecco's Modified Eagle Medium (DMEM) supplemented with 10% Fetal Bovine Serum (FBS), 1 mM HEPES, and 2 mM Glutamax. Madin-Darby Canine Kidney (MDCK) cells were cultured in DMEM supplemented with 10% FBS and 25 mM HEPES. P815 cells used as target cells for the CTL assay were cultured in DMEM media supplemented with 10% FBS. Mouse TC-1 cells were grown in RPMI media supplemented with 10% FBS and 10 mM HEPES.

### Design of universal influenza A H1 HA2 sequence and generation of recombinant adenoviruses

A universally conserved consensus sequence of the HA2 subunit of influenza A H1 was identified using a bioinformatics approach described previously [31]. Briefly, generation of the consensus sequence was accomplished by first downloading the set of all Influenza A H1 subtype HA sequences from the NCBI Flu database with the following filters: Flu A, all N subtypes, all hosts, all countries and all subtypes. The sequences were further filtered by removing ambiguities and redundancies. Sequences with ambiguous codons (BJOUXZ) were removed prior to alignment with Clustal-W. Aligned results were trimmed to the 226 residue HA2 region of interest before calculating consensus using the EMBOSS software package.

**Figure 1.**
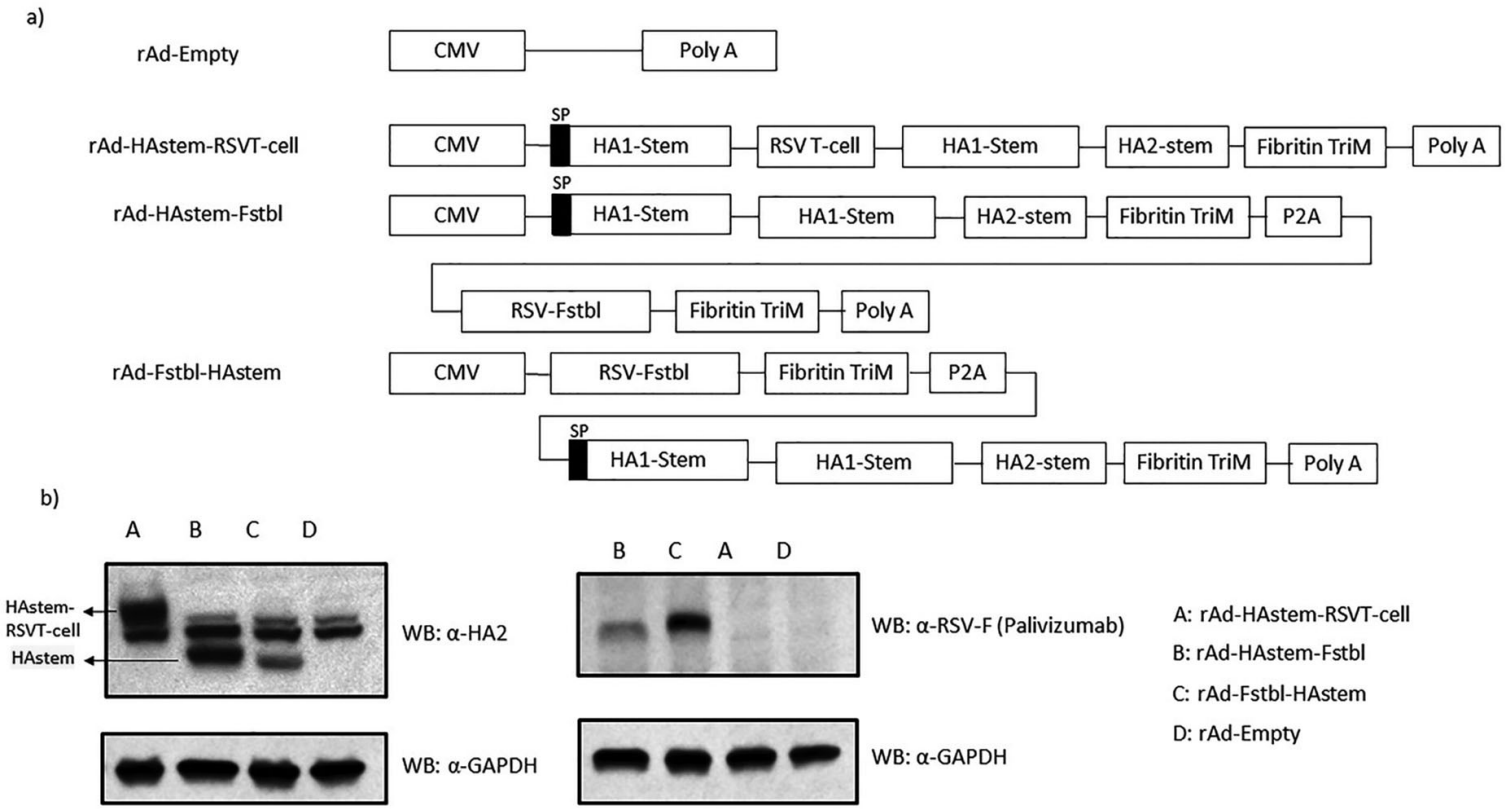
Recombinant adenovirus constructs and *in vitro* protein expression. (a) Schematic representation of the recombinant adenovirus (rAd) constructs. All the constructs were designed to express the HA stem domain, which contains the tyrosinase signaling peptide (SP) at the N-terminus of HA1-stem. (b) *In vitro* protein expression in mouse cell line. TC-1 mouse lung cells were infected with rAds and cell lysates were collected. Protein expression was confirmed using anti-HA2 polyclonal and anti-RSV-F (palivizumab) monoclonal antibodies. The line arrows indicate the HAstem and HAstem-RSVT-cell protein bands detected by the anti-HA2 antibody.

Recombinant adenoviruses were designed to express a trimeric secreted form of the consensus HA2 by including 23 amino acids (a.a.) from the human tyrosinase signal peptide (SP) at the N-terminus and a T4 fibritin trimerization motif (Fibritin TriM) as described previously [17]. The HA stem domain was designed by including the H1 HA2 consensus sequence (226 a.a.) described previously and two separate fragments from HA1 subunit of influenza B/Florida/04/06 (a.a. 16-69 and 306-361) to the N-terminus of HA2. The two HA1 stem fragments were separated by a GSGSG linker. The gene sequence for the Fstbl protein is derived from the F gene sequence of RSV subtype A strain (RSV-A2) by introducing stabilizing mutations as previously described [21]. In the rAd-HAstem-RSVTcell construct, five peptide fragments corresponding to six T-cell epitopes (Table 1) separated by GSGSG linkers were inserted between the two HA1 fragments of the HAstem sequence. The rAd-HAstem-Fstbl and rAd-Fstbl-HAstem constructs contained the prefusion stabilized RSV-F (Fstbl) separated by a Porcine Teschovirus-1 2A (P2A) [32] in two different orientations as shown in Figure 1A. The HAstem-Fstbl, Fstbl-HAstem and HAstem-RSVTcell sequences were synthesized by Bio Basic Canada Inc. The rAds were generated using the Gateway-adapted Virapower adenoviral expression system (Life Technologies) as described previously [17].

**Table 1.** T-cell epitope sequences included in the design of rAd-HAstem-RSVTcell.

#	T-cell epitope	Sequence inserted between the HA1 fragments
1	CD8 M2-1_(82-90)_	LGVVGVLESYIGSINNITKQSACVA
2	CD8 M2-1_(127-135)_	ELNSPKIRVYNTVISYIESNRKNNK
3	CD4 G_(181-197)_	CSICSNNPTCWAICKRIPNKKPGKKTTTKPTKK
4	CD4 N_(252-268)_	AGLFMNAYGAGQVMLRWGVLAKSVKNIMLGHAS
5,6	**CD8 F_(52-59)_** & CD4 F_(51-66)_	GYLSALRTG**WYTSVITI**ELSNIKENKCNGTDA

The underlined sequence represents the T-cell epitope sequence #5,6 encompasses both CD8 and CD4 sequences from F protein. CD8 epitope sequence is in bold and underlined, while CD4 epitope sequence is underlined.

### Animal studies

All animal procedures were approved by the Animal Care Committee at Health Canada, Ottawa. Six-week-old female Balb/c mice (Charles River, Saint Constant, QC) were immunized intranasally with 10^9^ PFU of the adenovirus constructs diluted in PBS. The vaccines were administered twice, over 28 days, and serum was collected on day 49 (21 days after the boost vaccination). For certain groups, spleen and lymph nodes (Tracheo-bronchial and Mediastinal) were collected in addition to blood at day 49 to isolate cells and measure cell-mediated immune responses. Mice were challenged with either 500 PFU or 1250 PFU of influenza A/Netherlands/602/09 (H1N1) or 3 × 10^6^ PFU of RSV-A2 (ATCC: VR-1540) at 28 days post boost immunization. Influenza A – challenged mice were weighed daily and monitored for signs of illness for 14 days. To determine viral burden, lungs and nose were collected following euthanasia at either three- or 5-days post-challenge from mice that were challenged with 500 PFU or 1250 PFU of influenza respectively. In the case of the RSV-challenged mice, the tissues were collected at 4 days post-challenge to assess viral burden and pathology.

### Western blotting

TC-1 mouse lung cells were infected with rAds at multiplicity of infection (MOI) of 600 and 48 h later, cell lysates were collected in RIPA lysis buffer. Lysates were electrophoresed on a SDS-PAGE gel and subsequently transferred to a polyvinylidene difluoride membrane. HAstem and RSV-F Protein expression was confirmed using anti-HA2 polyclonal that was generated in-house in New Zealand White Rabbits against a conserved epitope of H1 HA2 and anti-RSV-F (Palivizumab) monoclonal antibodies respectively.

### ELISA

The day 49 serum from immunized mice were used to determine influenza HA2- and RSV-F-specific IgG, IgG1 and IgG2a titers. Briefly, 96-well plates were coated with recombinant RSV-F protein (Sino Biological) at 0.5 µg/ml or recombinant influenza HA2 (rHA2) at 2 µg/ml diluted in PBS and coated overnight at 4°C. The rHA2 protein is derived from the HA sequence of influenza A/California/05/2009 (H1N1). It contains the same amino acid sequence of the HA stem construct M6EL(SS2) designed by Lu et al. [8]. The protein was expressed in *Escherichia coli* and purified in-house*.* After coating, ELISA was performed as described previously [17].

### Plaque assay

The plaque assay on the lungs from RSV-challenged mice and influenza A-challenged mice was performed as described elsewhere [17,33]. Briefly, the right lobe of the lung from RSV-challenged mice was collected at 4 days post-infection in serum free RPMI media. The tissues were weighed and homogenized using a high-speed mechanical homogenizer. The homogenates were clarified using centrifugation and serial dilutions of the clarified supernatant were overlaid on HEp-2 cells and incubated for 2 h at 37°C. At 2 h of incubation, the inoculum was removed and a 1:1 overlay of 2×DMEM media with 0.8% agarose was added. After 7 days of incubation, the overlay was removed, and the plaques were visualized by staining with crystal violet solution.

In the case of influenza-challenged mice, the right lobe of the lungs and nose were collected at 3- or 5-days post-infection and homogenized in PBS. MDCK cells were then incubated with serial dilutions of the clarified homogenates and incubated at 37°C for 1 h and 30 min. The inoculum was removed and the cells were overlaid with a 1:1 mixture of 2×DMEM and 1.6% agarose containing BSA and TPCK-treated trypsin at a final concentration of 0.2% and 2 µg/ml respectively. The overlay was removed following 3 days of incubation at 37°C and plaques were visualized by staining with a crystal violet solution.

### Microneutralization

The RSV-neutralizing ability of the serum from vaccinated mice was determined as described previously [33]. Briefly, 800 PFU of RSV-A2 was mixed with serial dilutions of the serum and incubated for 1hr at 37°C to facilitate the binding of serum antibodies to the virus. The mixture was then added to HEp-2 cells plated in 96-well plates and incubated at 37°C for 72 h. After 72 h, the cells were fixed and stained with HRP-conjugated anti-RSV antibody. The plates were then incubated with Tetramethylbenzidine (TMB) substrate and read spectrophotometrically at 450 nm.

Microneutralization assay for Influenza was carried out as described in the 2011 version of the WHO Manual for the laboratory diagnosis and virological surveillance of influenza [34]. Briefly, serum samples were treated with receptor-destroying enzymes (RDE) (Denka Seiken Co.) for 18 h at 37°C, followed by a 30-min incubation at 56°C. Serial dilutions of the serum were mixed with 100 TCID_50_ of the influenza A/Netherlands/602/09 (H1N1) virus and incubated for 1hr at 37°C. The mixture was then added to MDCK cells plated in 96-well plates and incubated at 37°C for 24 h. The cells were then fixed and the viral NP antigen was detected by indirect ELISA using a anti-influenza A NP antibody. The plates were then incubated with Tetramethylbenzidine (TMB) substrate and read spectrophotometrically at 450 nm.

### Antibody-dependent cellular cytotoxicity (ADCC)

The ADCC activity of the serum antibodies from vaccinated animals against RSV and influenza was measured as described previously [17,35]. To measure serum ADCC activity against influenza, 5 × 10^4^ MDCK cells infected with 5 MOI of influenza A/Netherlands/602/09 (H1N1) for 24 h were used as target cells. To measure serum ADCC against RSV, 2 × 10^4^ HEp-2 cells infected with 5 MOI of RSV-A2 were used as target cells. In both cases, serum samples were heat-inactivated, serially diluted and added to the infected cells. Then, Mouse FcγRIV effector cells were added and plates were incubated for 5 h at 37°C. The reporter activity from the effector cells was measured using Bio-Glo luciferase assay substrate (Promega Inc.) and luminescence values were recorded.

### Multiplex elisa to assess cytokine secretion

Splenocytes and lymph node cells were incubated with 5 µg/ml of each of the peptide (Table 1) in a pool or as individual peptides in a 96-well plate and incubated in a 5% CO_2_ incubator at 37°C for 24 h. As controls, cells were also incubated with DMSO in some of the wells. At 24 h, the media was collected and the secreted cytokines were measured using the Th1/Th2 Cytokine 11-Plex Mouse ProcartaPlex panel (ThermoFisher Scientific Inc.), following the manufacturer's recommended protocol.

### *In vitro* cytotoxic T lymphocyte (CTL) assay

The CTL assay was performed as described previously [36]. Briefly, 2 × 10^7^ splenocytes from mice were cultured for 5 days in a 5% CO_2_ incubator at 37°C in RPMI supplemented with 10% FBS and 0.1 µg/ml IL-2. The splenocytes were cultured either with 1 µg/ml of each of the three CD8 epitope peptides or with DMSO. At 5 days post-incubation, P815 target cells were pulsed with 10 µg/ml of each of the three CD8 epitope peptides or treated with DMSO for 2 h. Stimulated and unstimulated splenocytes were then plated on 96-well plates and 2-fold serial dilutions of the cells were done across the 96-well plate. The splenocytes were then co-cultured with either peptide-pulsed P815 cells or DMSO-treated P815 cells and incubated in a 5% CO_2_ incubator at 37°C for 4 h. At 4 h of incubation, the LDH released in the media was measured using a CyQUANT LDH cytotoxicity assay kit (Thermofisher Scientific Inc.) and the percentage of peptide-specific lysis was calculated.

### Pathology

Left lung lobes from the mice were inflated and fixed in 10% neutral buffered formalin. The lung lobes were further processed and lesions scored for pathology as described previously [17]. Briefly, the lung lobes were trimmed transversely to generate four slices from different areas of the lobe and were processed and embedded into paraffin blocks. Four micron thick sections were produced and stained with Harris hematoxylin (Thermofisher Scientific Inc.) and instant eosin (Thermofisher Scientific Inc.) using a Gemini AS autostainer.

The lung lesions were classified using the Society of Toxicologic Pathology's International Harmonization and Diagnostic criteria (INHAND) for the respiratory tract [37]. A subjective numeric lesion severity score was given, where 0 means the lesion is absent; 1 means minimal; 2 means mild; 3 means moderate; 4 means marked; 5 means severe. Blood vessel changes were classified using the INHAND guideline for the cardiovascular system [38]. Mice that received PBS only and had no significant lesions were used as negative controls for scoring purpose.

### Statistical analysis

Measurements were taken from distinct samples in all assays tested. Normality of the study data was assessed by a Shapiro–Wilk normality test. For the data deemed not of normal distribution, non-parametric approaches, Kruskal Wallis/Wilcoxon Rank Sum Test were applied; the *p*-values of non-parametric tests for the multiple pairwise comparisons (each of vaccinated groups vs. control) were adjusted using step-down Bonferroni (Holm) method. An analysis of variance (ANOVA) was applied for normally distributed data; a Tukey's *post hoc* or Dunnet's *post hoc* test was applied for the multiple comparisons for the study outcomes with significant ANOVA result. A log-rank test was applied for the censored (survival) data. To investigate if a vaccinated group is superior to the control group, a one-sided hypothesis testing was applied with a significance level of 0.05 for pairwise statistical tests. The statistical analyses were conducted using GraphPad Prism 7 and SAS Enterprise Guide 7.1 software.

## Results

### Construction of recombinant adenoviruses and protein expression

We generated recombinant adenoviruses (rAd) encoding the gene sequence for codon optimized, trimeric, secreted form of influenza A HA stem region (HAstem) and either the gene sequence of pre-fusion stabilized RSV-F (Fstbl) or several of known immunodominant and subdominant RSV CD8 and CD4 T-cell epitopes (Figure 1a and Table 1). The HA stem region consists of a HA2 consensus sequence generated using all known hemagglutinin sequences of the H1 subtype influenza A from NCBI and two separate fragments from the HA1 subunit of influenza B/Florida/04/06 (amino acids 16–69 and 306-361). The HA2 consensus sequence was created as described previously [17]. An adenovirus construct carrying no foreign antigen sequence (rAd-empty) served as the control. *In vitro* protein expression upon infection with the adenovirus constructs was confirmed by Western blot (Figure 1b).

### Recombinant adenovirus vaccines induce high levels of antigen-specific serum antibodies with effector functions

To evaluate the efficacy of the vaccine constructs, mice were immunized intranasally with 10^9^ PFU of adenovirus in a prime/boost regimen 4 weeks apart and investigations were performed in the sera collected 3 weeks post the boost vaccination. We observed significantly high levels of HA2-specific IgG, IgG_1_ and IgG_2a_ antibodies in all three vaccinated groups (Figure 2a). Notably, levels of IgG_2a_ in the serum were higher than IgG1 antibodies, leaning towards Th1-biased immune response (Figure 2b). Similarly, the vaccines induced significantly high levels of antibodies against RSV-F protein, except for the vaccine carrying the RSV T-cell epitopes (rAd-HAstem-RSVTcell), where the levels of anti-RSV-F antibodies were either undetectable or not different than the rAd-empty vaccinated control group (Figure 2c). Similar to the immune response against Influenza A, the IgG_2a_:IgG_1_ ratio denoted an inclination for a Th1-biased immune response (Figure 2d). Figure 2.Vaccination induced robust production of anti-HA2 and anti-RSV-F serum antibody titers. (a) Influenza HA2-specific circulating IgG, IgG1 and IgG2a antibodies at 3 weeks after boosting are shown (*n* = 5). Statistical analysis performed using Kruskal Wallis (non-parametric) test. Data shown is mean ± SEM, **p < 0.01. (b) HA2-specific serum IgG2a:IgG1 ratio indicating Th2- or Th1-biased nature of the immune response (*n* = 5). Dotted line denotes the threshold (y = 1) for determining immune responses as Th1 or Th2 biased. Statistical analysis performed using one-way ANOVA with Tukey's *post hoc* test. Data shown is mean ± SEM. ns = not significant. (c) RSV F-specific circulating IgG, IgG1 and IgG2a antibodies at 3 weeks after boosting are shown (*n* = 5). Statistical analysis performed using Kruskal Wallis (non-parametric) test. Data shown is mean ± SEM, **p < 0.01. (d) RSV F-specific serum IgG2a:IgG1 ratio indicating Th2- or Th1-biased nature of the immune response (*n* = 5). Dotted line denotes the threshold (y = 1) for determining immune responses as Th1 or Th2 biased. Statistical analysis was performed using paired *t*-test. Data shown is mean ± SEM. ns = not significant.

**Figure:**
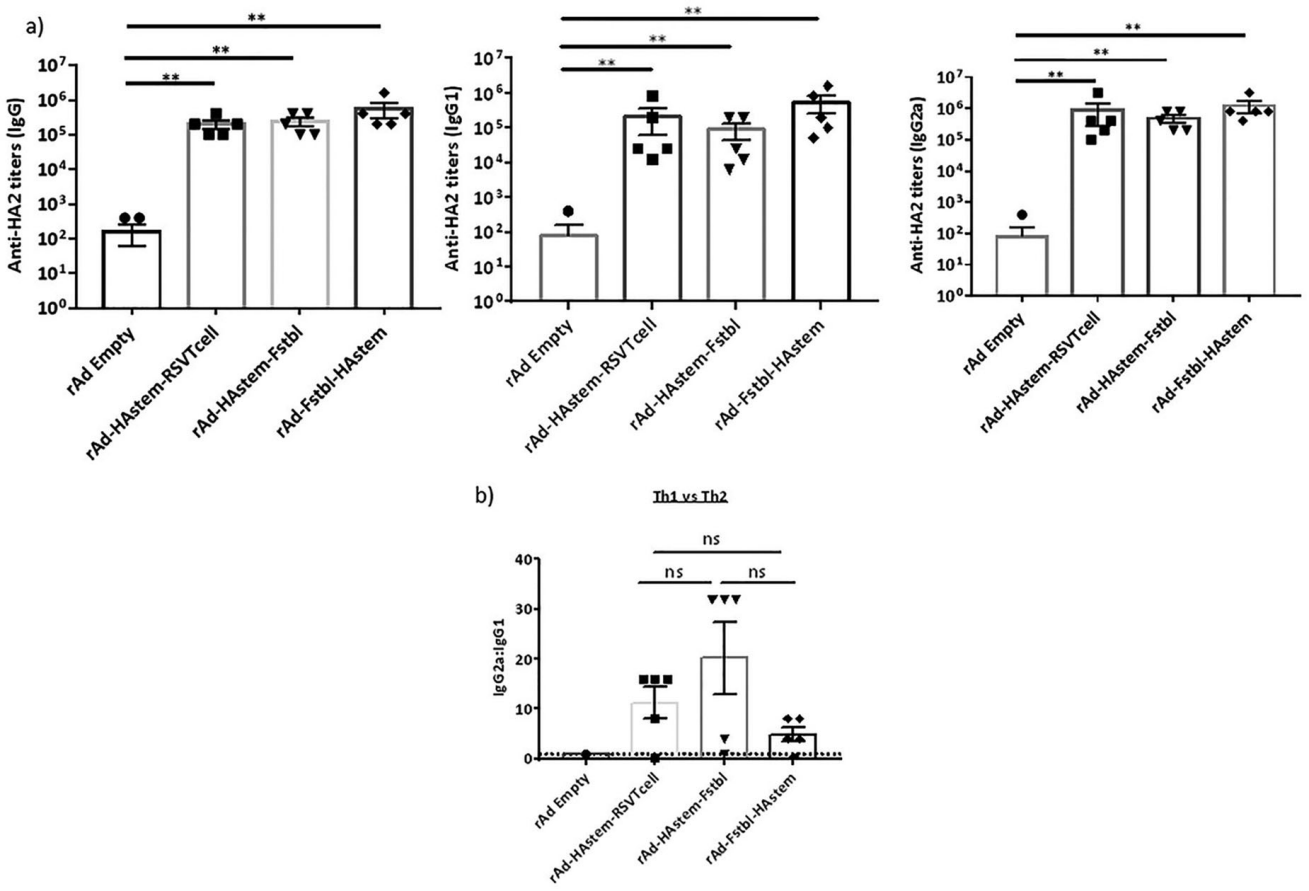

**Figure:**
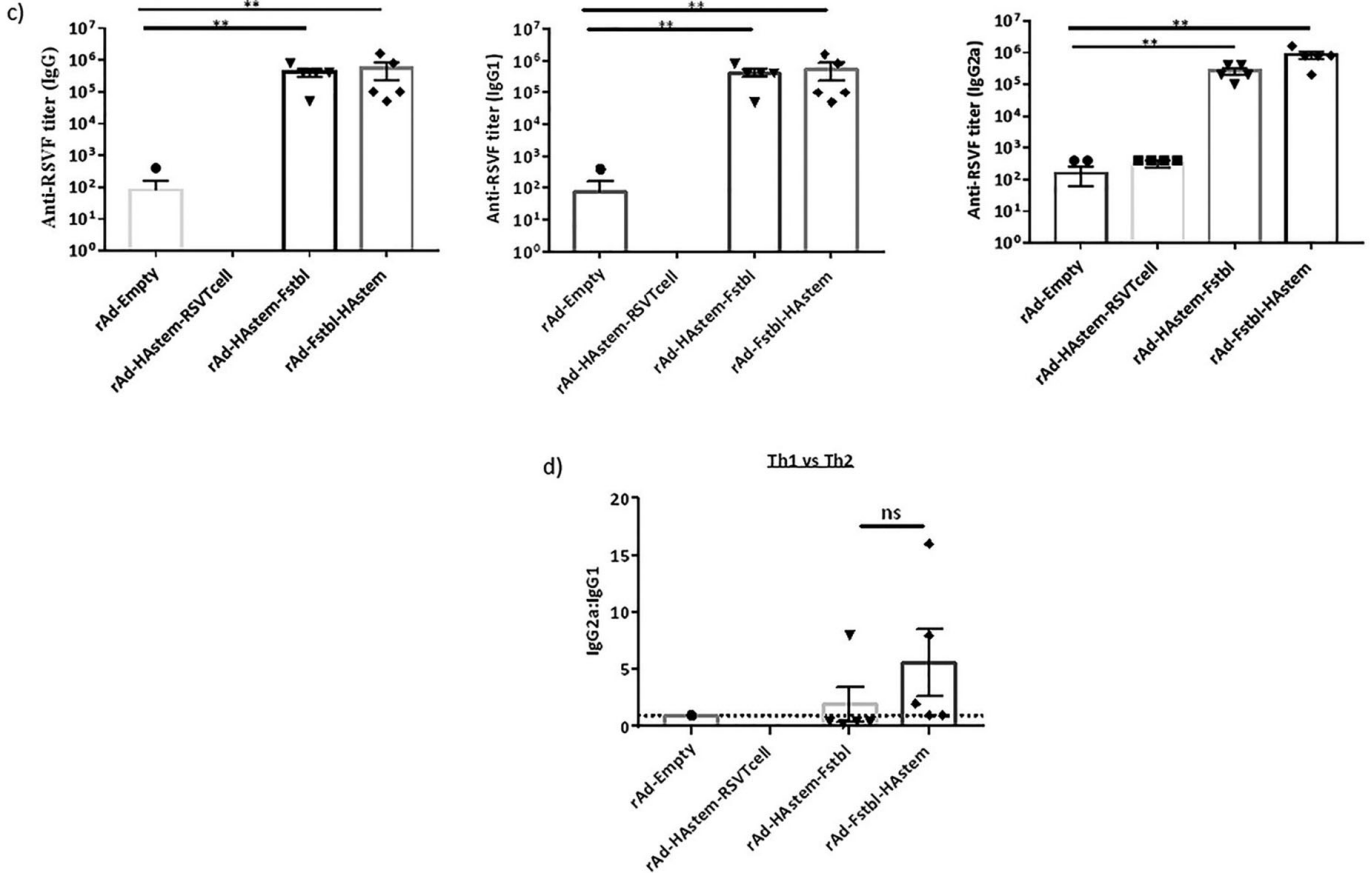

**Figure 3.**
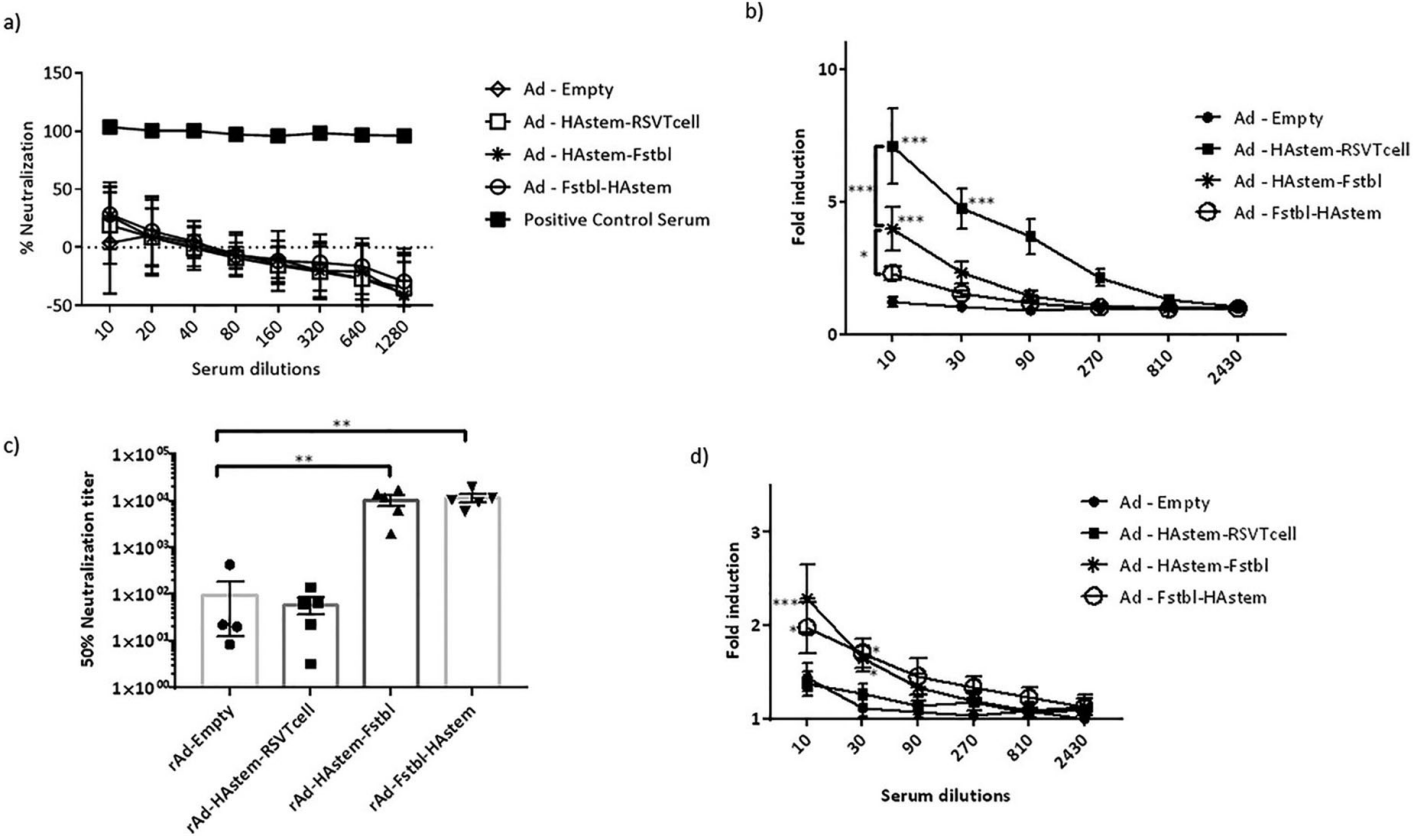
Serum antibodies possess neutralizing and ADCC effector functions against influenza and RSV. (a,b) Serum collected post-boosting was used to determine the neutralizing ability (a) and ADCC activity (b) against influenza A/Netherlands/602/09 (H1N1). Anti-Influenza A/California/7/09 HA serum (NIBSC code – 14/310) was used as a positive control for the neutralization assay. RSV-A2 neutralizing ability (c) and ADCC activity against RSV-A2 (d) of the mice serum collected post-boosting (*n* = 5) is shown. (b,d) Fold induction over “no antibody” control is shown. Data shown is mean ± SEM, *p < 0.05, **p < 0.01, ***p < 0.001. (two-wayANOVA with Tukey's *post hoc* test (b), paired *t*-test (c), Dunnett's *post hoc* test (d)).

Antibodies exert their effector functions by various mechanisms, including neutralization of pathogen and Antibody-dependent cellular cytotoxicity (ADCC) [39,40]. IgG2a subclass is a potent activator of ADCC [41]. Therefore, we performed a microneutralization assay and ADCC assay against both influenza and RSV. Consistent with our previous study on a HAstem vaccine against influenza B, we did not observe any neutralizing antibodies against influenza in the serum from vaccine-boosted animals (Figure 3a) [17]. However, significantly high levels of serum antibodies with ADCC activity against influenza were observed in animals vaccinated with rAd-HAstem-RSVTcell and rAd-HAstem-Fstbl (Figure 3b). Interestingly, the serum from mice vaccinated with the HAstem-RSVTcell construct had significantly higher ADCC activity compared to the serum from HAstem-Fstbl and Fstbl-HAstem vaccinated animals (an increase of 1.8 fold and 2.4 fold respectively), at a serum dilution of 1:10. Moreover, vaccination with constructs containing the pre-fusion stabilized F gene sequence (Fstbl) and not the RSV T-cell epitopes induced significantly high levels of neutralizing antibodies (Figure 3c) and ADCC-active antibodies against RSV (Figure 3d).

### Immune cells from mice vaccinated with constructs containing RSV T-cell epitopes possess peptide-specific activation and CTL activity

As vaccination with the vector encoding RSV T-cell epitopes does not induce antibodies that have RSV-specific neutralizing or ADCC effector functions (Figure 3), we assessed the induction of cell-mediated immune response in these mice. Stimulation of both splenocytes and lymph node cells with a pool of the peptides corresponding to the six T-cell epitopes included in the construct, induced strong RSV-specific secretion of several cytokines at 24 h post-stimulation (Figure 4a). Although both Th1 and Th2 cytokines were secreted, the expression levels were remarkably high for Th1 cytokines such as IFNγ, IL2, and TNFα. Whereas IL-18 expression can drive either a Th1 or Th2 response depending on the cytokine milieu, the high levels of IFNγ indicates the promotion of a Th1 response by IL-18. Stimulation with individual peptides, showed a clear dominance of the CD8 M2-1 epitopes (Peptides 1 & 2) followed by the CD4 G and N epitopes (Peptides 3 & 4), which is consistent with the findings reported by Varga et al., (Figure 4b). Furthermore, we confirmed the CTL functionality of CD8-epitope peptide-stimulated splenocytes as they were effectively able to lyse CD8-epitope peptide-pulsed target P815 cells *in vitro* (Figure 4c). Clearly, insertion of RSV T-cell epitope sequences in the influenza HA could elicit a RSV-specific cell-mediated immune response. Figure 4.RSV CD4 and CD8 epitope peptide stimulation induces cytokine secretion from immune cells isolated from rAd-HAstem-RSVTcell vaccinated mice. (a) Splenocytes and Lymph node cells were incubated with a pool of six T-cell epitope peptides at 5 µg/ml each (*n* = 5). A panel of Th1/Th2 secreted cytokines were measured at 24 h of incubation. Data shown is mean ± SEM. (b) Splenocytes and Lymph node cells were incubated with each of the six T-cell epitope peptides (Table 1) at 5 µg/ml (*n* = 5). A panel of Th1/Th2 secreted cytokines were measured at 24 h of incubation. Data shown is mean ± SEM. Statistical data corresponds to comparison of vaccinated and control mice. (c) Splenocytes from vaccinated animals were stimulated with a pool of the three CD8 epitope peptides for 5 days and were co-incubated with peptide-pulsed P815 target cells for 4 h (*n* = 5). Peptide-specific target cell lysis was calculated based on the amount of LDH released in the media. Data shown is mean ± SEM. *p < 0.05, **p < 0.01, ***p < 0.001 (*t*-tests (a,b,c))

**Figure:**
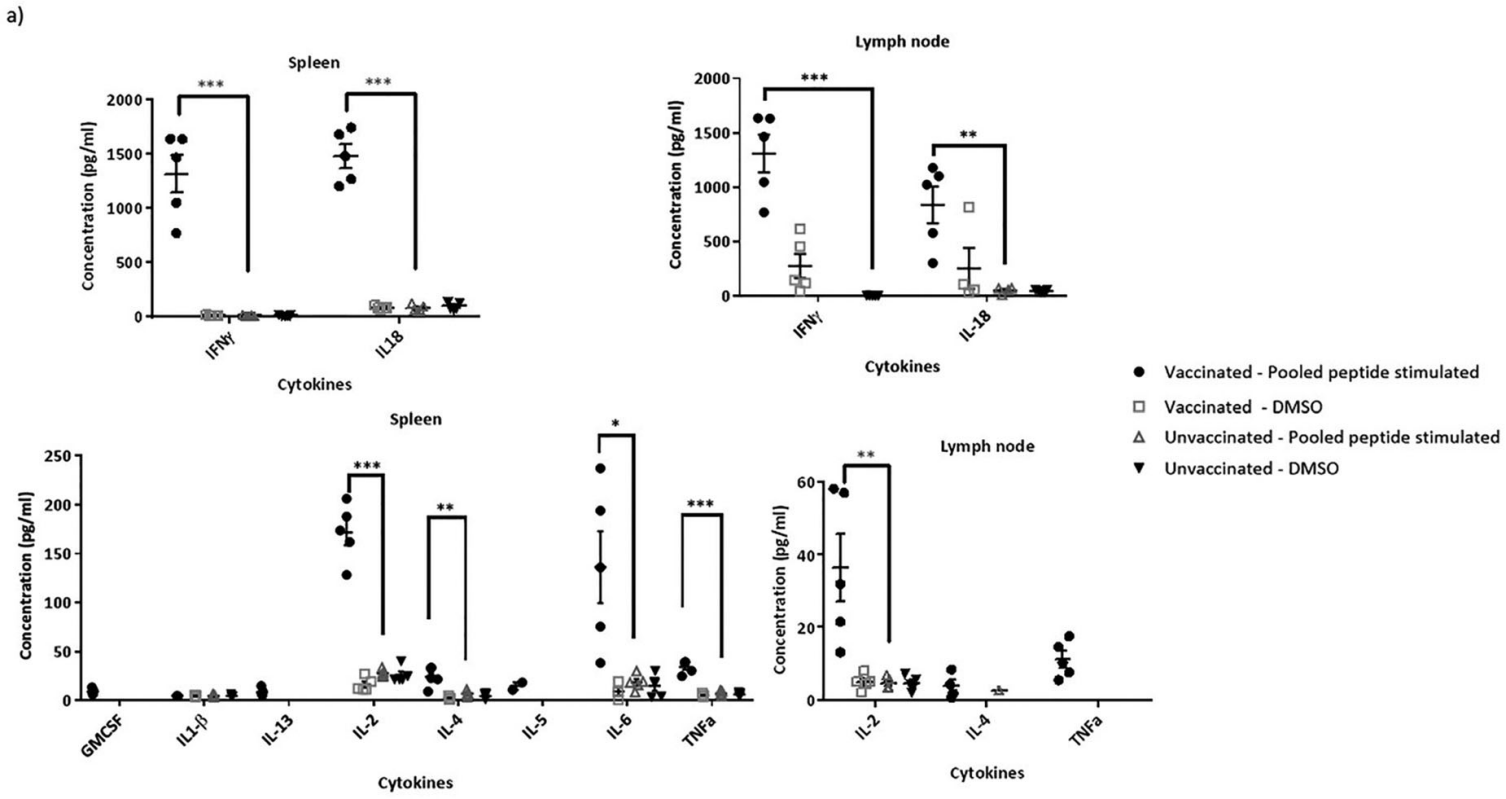

**Figure:**
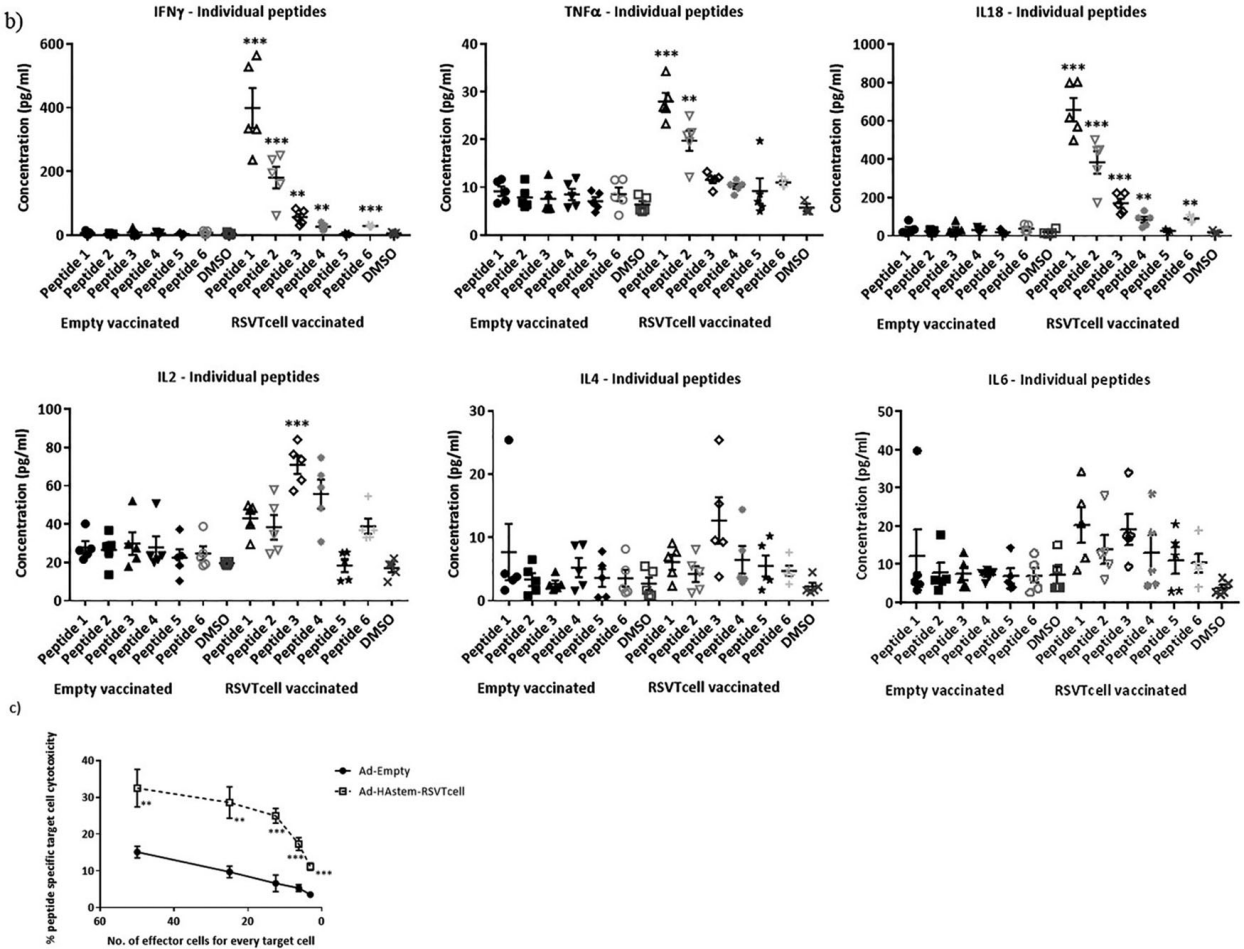

### The adenovirus constructs afford significant protection against both influenza and RSV

**Figure 5.**
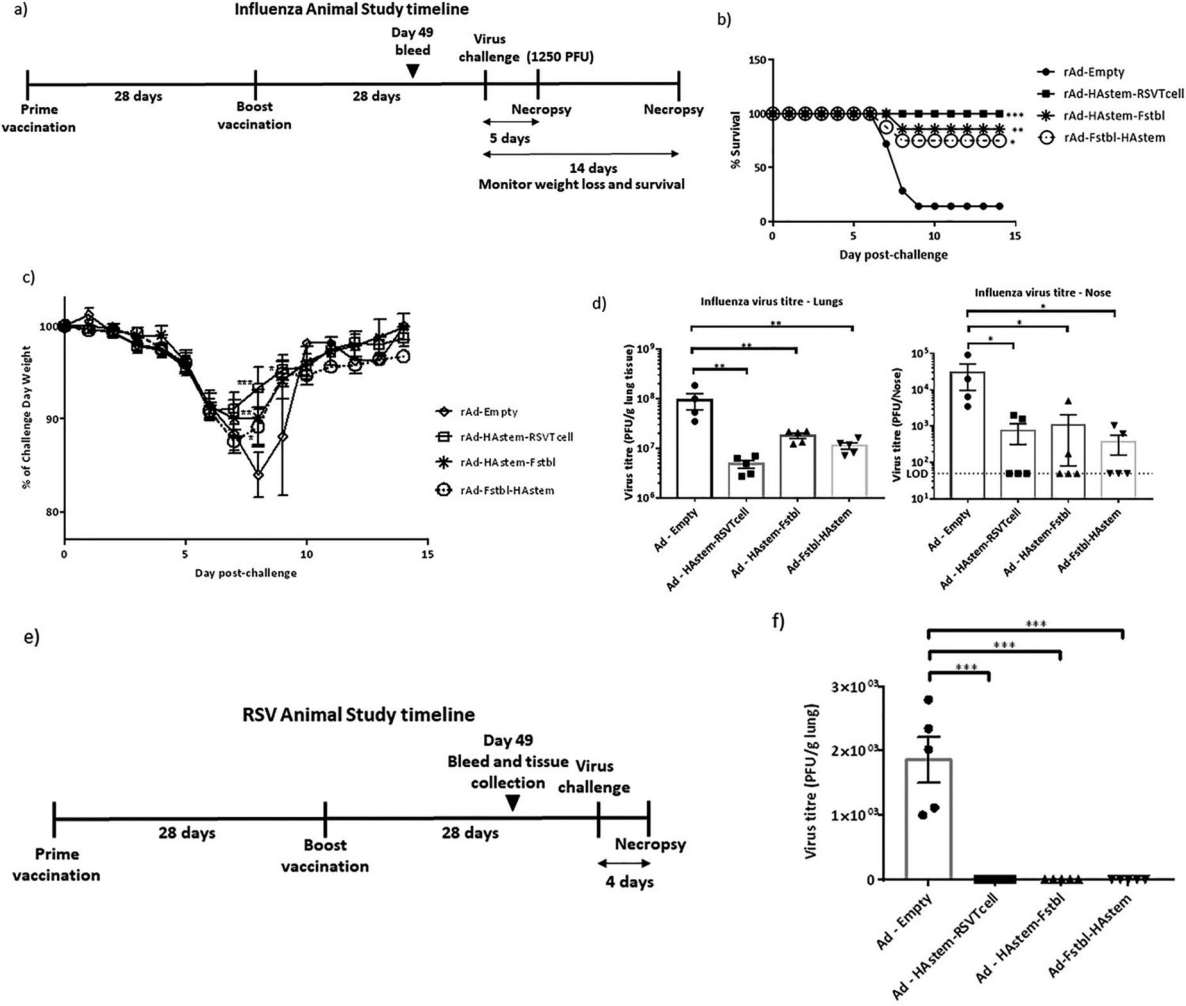
Intranasal immunization with the vaccine constructs provide significant protection against influenza and RSV challenge. (a) Schematic diagram of the immunization, influenza A challenge and necropsy timeline at a challenge dose of 1250 PFU/mouse. (b–d) BALB/c mice were vaccinated intranasally in a prime/boost regimen, 4-weeks apart, with the rAd vaccines and challenged intranasally with 1250 PFU/mouse of influenza A. (b) Survival (*n* = 7 or 8) is shown. Median survival time since day of challenge – Ad-empty: 7 days, all vaccinated groups: > 14 days. *p < 0.05, **p < 0.01, ***p < 0.001, log-rank test. (c) Weight loss data is shown. Data shown is mean ± SEM. (d) Virus titre from infected lungs and nose collected 5 days post-challenge were determine by plaque assay (*n* = 4 or 5). Data shown is mean ± SEM. The dotted line denotes the limit of detection (LOD) of the assay. *p < 0.05, **p < 0.01, ***p < 0.001 (two-way ANOVA (Tukey's *post hoc* test) (c), one-way ANOVA (Dunnet's *post hoc* test) for lung viral titers and Kruskal-Wallis (non-parametric) test for the Nose viral titers (d). (e) Schematic diagram of the immunization, RSV-A2 challenge and necropsy timeline. (f) BALB/c mice were vaccinated intranasally in a prime/boost regimen, 4-weeks apart, with the rAd vaccines and challenged intranasally with RSV-A2. Virus titre from infected lungs collected 4 days post-challenge were determine by plaque assay (*n* = 5). Data shown is mean ± SEM. ***p < 0.001.one-way ANOVA (Dunnet's *post hoc* test).

To evaluate the efficacy of the vaccine constructs in protecting against an influenza infection, immunized mice were challenged with two different doses of influenza, a lower dose of 500 PFU/mouse (Figure S1a) or a higher dose of 1250 PFU/mouse (Figure 5a). We chose two different challenge doses in order to delineate possible differences in the protective efficacy among the three vaccine constructs. Challenge at 500 PFU was sub-lethal, resulting in 50% of mice reaching euthanasia end-point in the control group (Figure S1b). Notably, while the control mice experienced significant loss of body weight (BW), vaccination protected the animals from BW loss and reduced nose viral burden (Figures S1c and S1d). However, the lung viral burden was not significantly reduced in the vaccinated animals when compared to the control (Figure S1d) at 3 days post challenge, which is likely due to this time point being too early to observe vaccine-induced effects on lung viral clearance. Therefore, we tested the effectiveness of the vaccine at a lethal challenge dose of 1250 PFU (Figure 5a) and assessed respiratory viral burden at a late time point of 5 days post challenge. Whereas the HAstem-RSVTcell vaccine afforded complete protection from mortality, the HAstem-Fstbl and Fstbl-HAstem vaccination had lower survival rates at 86% and 75% respectively. However, vaccination with all three vaccines improved survival of the animals significantly when compared to Ad-Empty vaccinated animals, where only 14% of the animals survived the challenge (Figure 5b). Moreover, at this higher challenge dose, the vaccinated animals suffered significantly lower BW loss at day 8 post-challenge than the control group (Figure 5c). The increase in survival correlated with viral burden, as HAstem vaccination resulted in a pronounced reduction of the viral titers in both the lungs and nose of infected mice (Figure 5d).

While we observed significant weight loss and death in the control group upon influenza challenge no significant BW loss or death was observed upon RSV challenge in any of the groups as expected (Figure S2), since mice are known to be semi-permissive to RSV infection [42]. However, all three vaccines were highly effective in protecting the mice against lung viral replication, as no detectable plaques were observed in the lungs of vaccinated and RSV-A2 challenged mice (Figure 5(e,f)).

### Vaccination reduces the severity of lung pathology caused by infection

We next assessed the ability of the vaccines to protect from infection-induced pathology in the lungs. Overall, both RSV and influenza induced mild pathology in the lungs of the control-vaccinated animals. While the inflammation in influenza-infected lungs was characterized by mild to moderate bronchiolitis and moderate to marked perivascular cuffing, the inflammation in RSV-infected lungs was characterized mostly by marked to severe perivascular cuffing. In the influenza challenged mice, although not statistically significant, vaccination appeared to lower the pathological scores when compared to vector control (Figure 6(a,b)). In the RSV challenged mice, when compared to the control, vaccination with adenoviruses encoding the Fstbl or RSV-T cell sequences, reduced overall severity of RSV-induced lung pathology to minimal-mild levels in the animals (Figure 6(c,d)). It is also worth noting that no vaccine-associated enhanced lung pathology was observed in any of the vaccinated groups. Figure 6.Pathological scoring of lung tissue following virus challenge. (a) Average pathological score of lung tissue from mice 5 days post-challenge with influenza A. (b) Representative images of H&E stained mice lungs post challenge with influenza at 20× magnification. Long arrows point to perivascular cuffing, short arrows point to epithelial cell hyperplasia and arrowheads point to necrotic epithelial cells. Length of the scale bar is 50 µm. (c) Average pathological score of lung tissue from mice, 4 days post-challenge with RSV-A2. (d) Representative images of H&E stained mice lungs post challenge with RSV at 20× magnification. Long arrows point to perivascular cuffing. Length of the scale bar is 50 µm. Data shown is mean ± SEM, *p < 0.05 (Kruskal Wallis (non-parametric) test).

**Figure:**
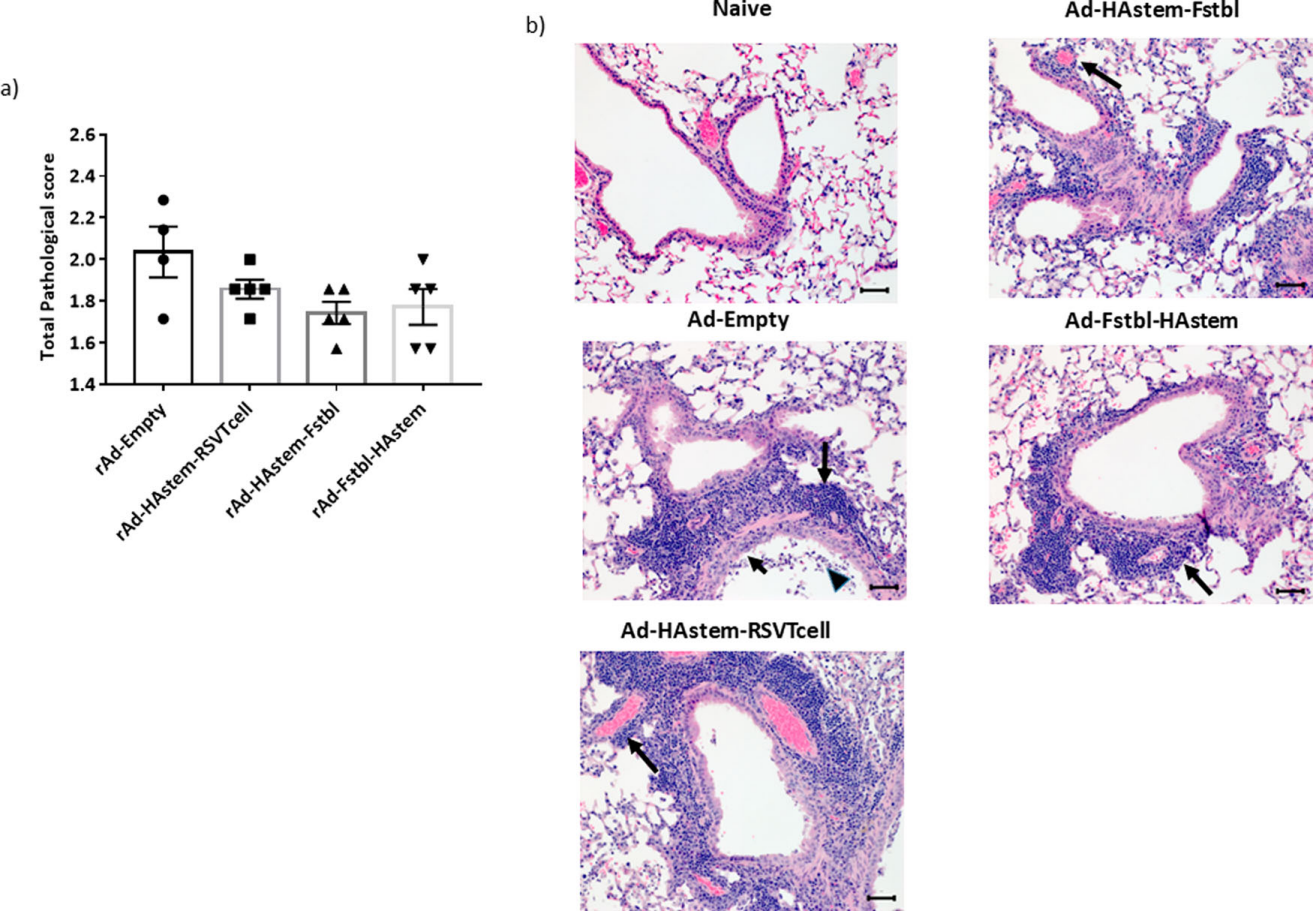

**Figure:**
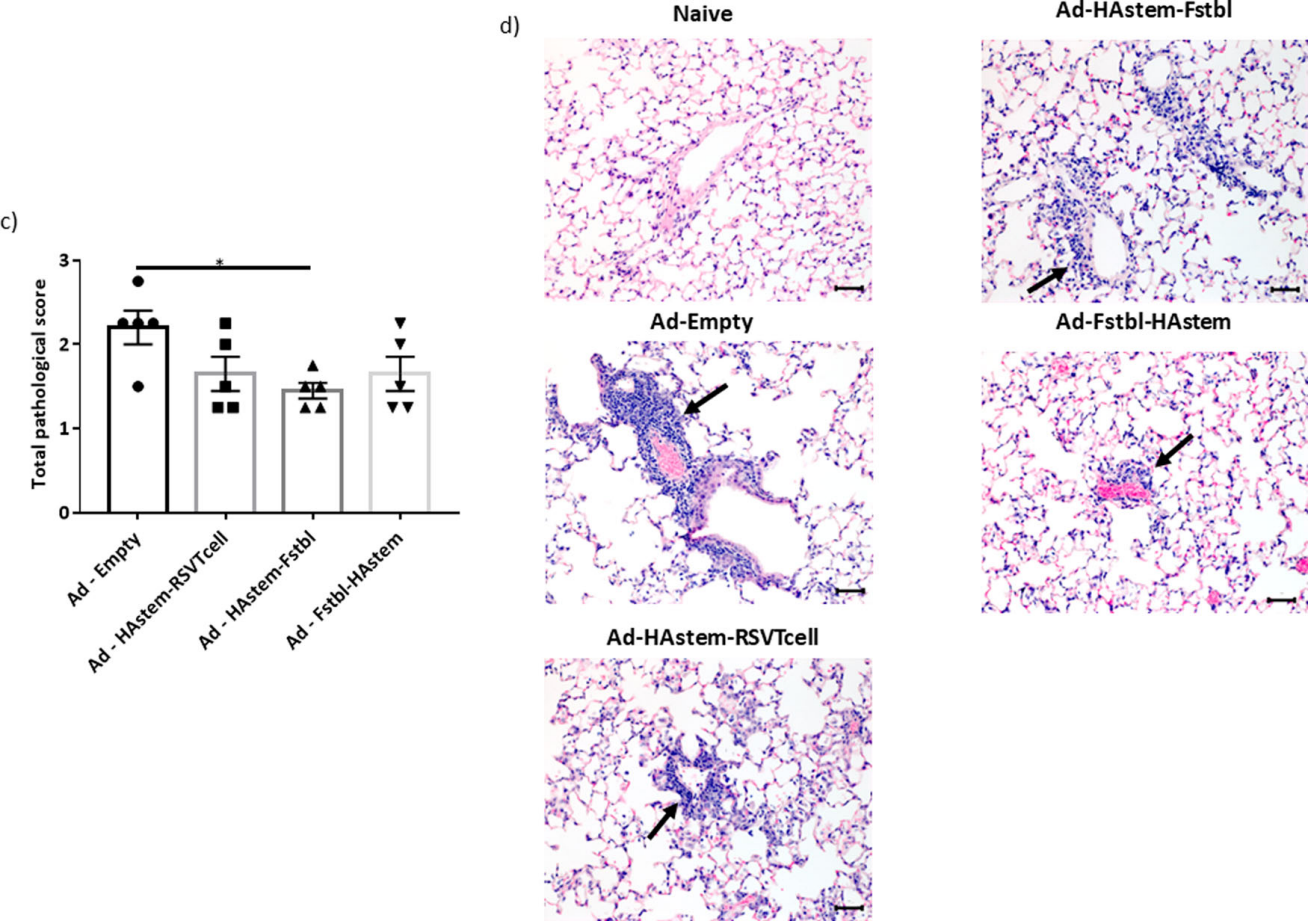

## Discussion

There are three specific aims in this current study. First, we intended to investigate whether the position of the RSV and influenza genes in the adenovirus construct could affect the expression levels and ultimately the level of protection, as gene positions can affect expression levels in bi-cistronic or poly-cistronic constructs [43]. Second, we wanted to test whether incorporation of immunodominant RSV T-cell peptide epitopes is sufficient to afford protection against RSV challenge and whether such an insertion, replacing the “Head” domain of HA affects the immune response elicited by HAstem. Third, we wished to shed light on the mechanisms underlying the protection induced by the bivalent vaccines.

To address the first question, we designed two constructs containing the HAstem and Fstbl antigens in two different orientations, HAstem-P2A-Fstbl or Fstbl-P2A-HAstem (Figure 1a). While the gene at the first position appeared to be favored for high expression levels (Figure 1b), no marked difference was observed in the survival, weight loss or respiratory viral load in the vaccinated animals upon challenge with either influenza A or RSV (Figure 5), results that are in agreement with all the serum antibody immune correlates (Figures 2 and 3). Though, we observed a minimal, albeit significant, difference in influenza ADCC activity at the 1:10 serum dilution among the HAstem-Fstbl and Fstbl-HAstem vaccine recipients (Figure 3b).

To address the second question, we replaced the “Head” of HA1 with the with RSV T cell epitopes to build the bivalent vaccine - rAd-HAstem-RSVTcell. Given that HA1 domain is necessary to maintain the native structure of the HA2 domain [8–13,44,45], any alterations of the HA1 region could affect HA2 conformation and subsequently the immune response. Indeed, we observed that the insertion of the RSV sequence in the influenza HA backbone induced augmented ADCC activity in mice vaccinated with rAd-HAstem-RSVTcell (Figure 3b). We speculate that certain HA2-ADCC epitopes previously unavailable in the HA2 stem antigen became exposed upon filling in the empty “Head” region of HAstem with RSV T cell epitopes. Clearly, the increased protection cannot be attributed to levels of HA2-specific IgG2a, as the titers are similar across all three vaccine constructs (Figure 2a).

To address the third question, we performed various assays to interrogate the mechanisms underlying vaccine-induced protection. Regarding influenza challenge, our data are in agreement with previous observations that ADCC and Fc receptor interactions of HA stem-specific antibodies are important mediators for protection against influenza [46–48]. In RSV challenge study, although antibodies with RSV neutralizing and ADCC effector functions were not induced by the bivalent vaccine (rAd-HAstem-RSVTcell), the vaccine did induce activation of RSV specific splenocytes and Lymph node cells, indicating a prominent role for cell-mediated immune responses (Figure 4a,b). It is worth noting that during RSV infection, the immunodominant CD8 T-cell epitope M2-1_(82-90)_ accounts for almost a third of the CD8 T cell responses, while the most immunodominant CD4 T-cell epitope accounts for only 2–3% of the total CD4 T-cell response [29]. It is interesting that this immunodominance hierarchy is retained in the immune response induced by our vaccines, even in the absence of the repertoire of viral proteins that are present during an active RSV infection (Figure 4b). In another study, where both M2-1_(82-90)_ and M2-1_(127-135)_ epitopes were inserted in the LAIV vector backbone as a vaccine, splenocytes from vaccinated animals were stimulated only by the M2-1_(82-90)_ peptide and not by the M2-1_(126-145)_ peptide [49]. However, in our study, we found that both the epitopes contribute to cytokine secretion upon stimulation of splenocytes (Figure 4b). This discrepancy could be due to differences in the length of the amino acid sequences flanking the core peptide epitope included in the vaccine design as well as the differences in the peptide used for stimulation. Finally, along with antiviral cytokines, the increased cytotoxic T-cell activities could be another important mechanism underpinning the bivalent vaccine-induced protection (Figure 4c).

In conclusion, our study is tailored to address specific questions outlined above. As a proof-of concept, bivalent vaccines described here could be effective against both influenza and RSV, as the antigens are highly conserved and proven to be capable of protecting against multiple strains [16,50,51]. The HAstem, as an antigen, can reduce viral replication and increase protection against both group 1 and 2 influenza A viruses and Victoria and Yamagata lineages of influenza B viruses [16,17]. Additionally, vaccines based on the RSV-F protein can offer heterologous protection against both subtypes of RSV and monoclonal antibodies targeting pre-fusion RSV-F epitopes are cross-reactive against a broad panel of RSV strains [50–53]. Although out of scope in this communication, other important questions including utility of molecular adjuvants such as CD40L, long term protection and challenge with more diverse virus strains, would need to be addressed along with other different antigen delivery systems. Nonetheless, considering the recent renewed interest from vaccine manufacturers in developing a combination vaccine against respiratory viruses such as RSV, influenza and SARS-CoV-2 [54,55], it is essential to develop and investigate vaccine designs and platforms that could confer such multi-valence.

## Supplementary Material

Supplemental Material

## Data Availability

Data have been deposited in the Government of Canada Centralized repertoire and would be available upon request after publications.
