# Supplementary material for: Bivalent vaccines effectively protect mice against influenza A and respiratory syncytial viruses
Source: Emerg Microbes Infect. 2023 May 9;12(1):2192821. doi: 10.1080/22221751.2023.2192821 (PMC10171128; doi:10.1080/22221751.2023.2192821)
Supplement: Supplemental Material [file TEMI_A_2192821_SM8901.pptx]

## Slide 1
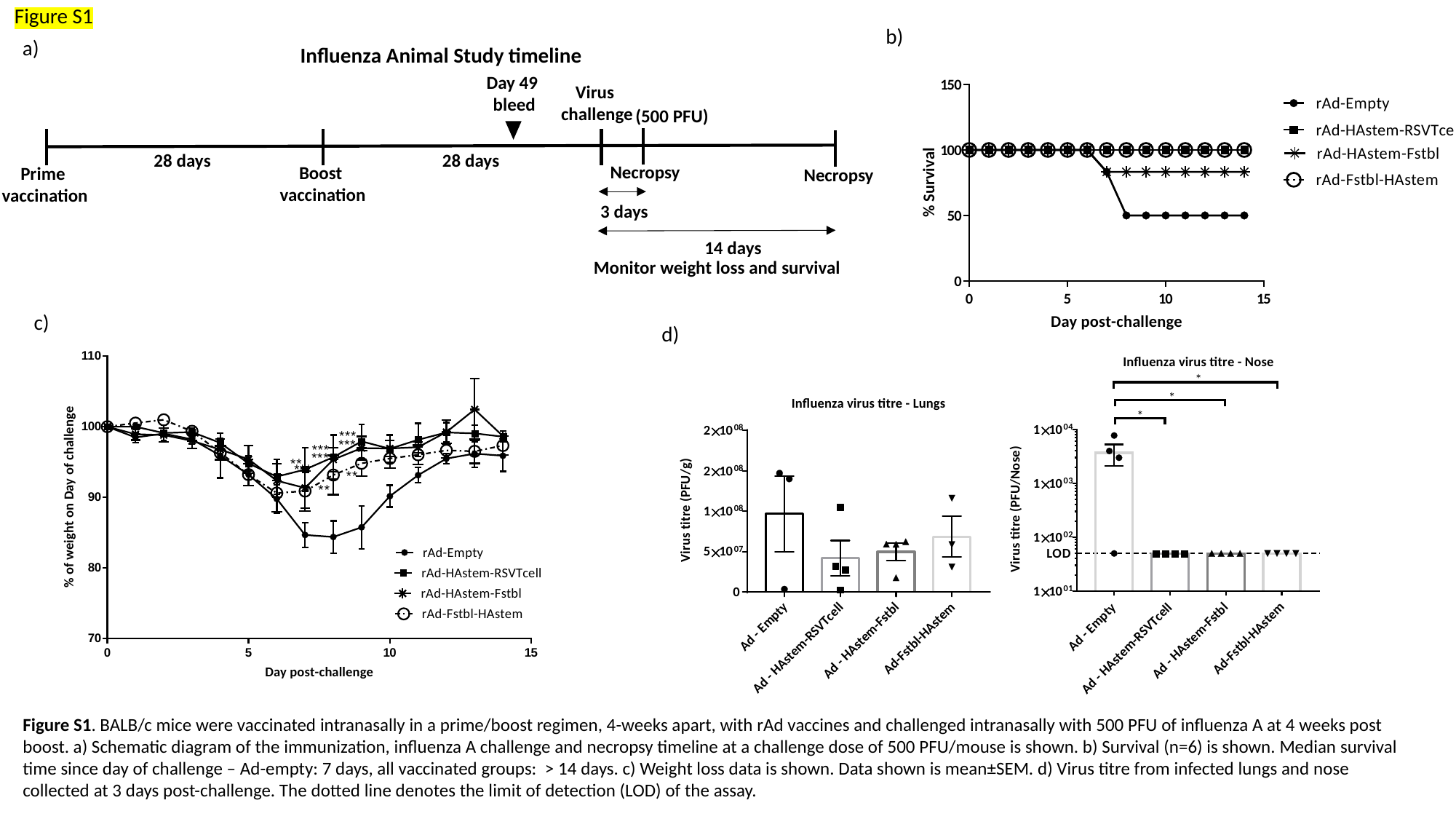

Figure S1
b)
a)
Influenza Animal Study timeline
Day 49
bleed
Virus
challenge
(500 PFU)
28 days
28 days
Necropsy
Boost
vaccination
Prime
vaccination
Necropsy
3 days
14 days
Monitor weight loss and survival
c)
d)
Figure S1. BALB/c mice were vaccinated intranasally in a prime/boost regimen, 4-weeks apart, with rAd vaccines and challenged intranasally with 500 PFU of influenza A at 4 weeks post boost. a) Schematic diagram of the immunization, influenza A challenge and necropsy timeline at a challenge dose of 500 PFU/mouse is shown. b) Survival (n=6) is shown. Median survival time since day of challenge – Ad-empty: 7 days, all vaccinated groups: > 14 days. c) Weight loss data is shown. Data shown is mean±SEM. d) Virus titre from infected lungs and nose collected at 3 days post-challenge. The dotted line denotes the limit of detection (LOD) of the assay.

## Slide 2
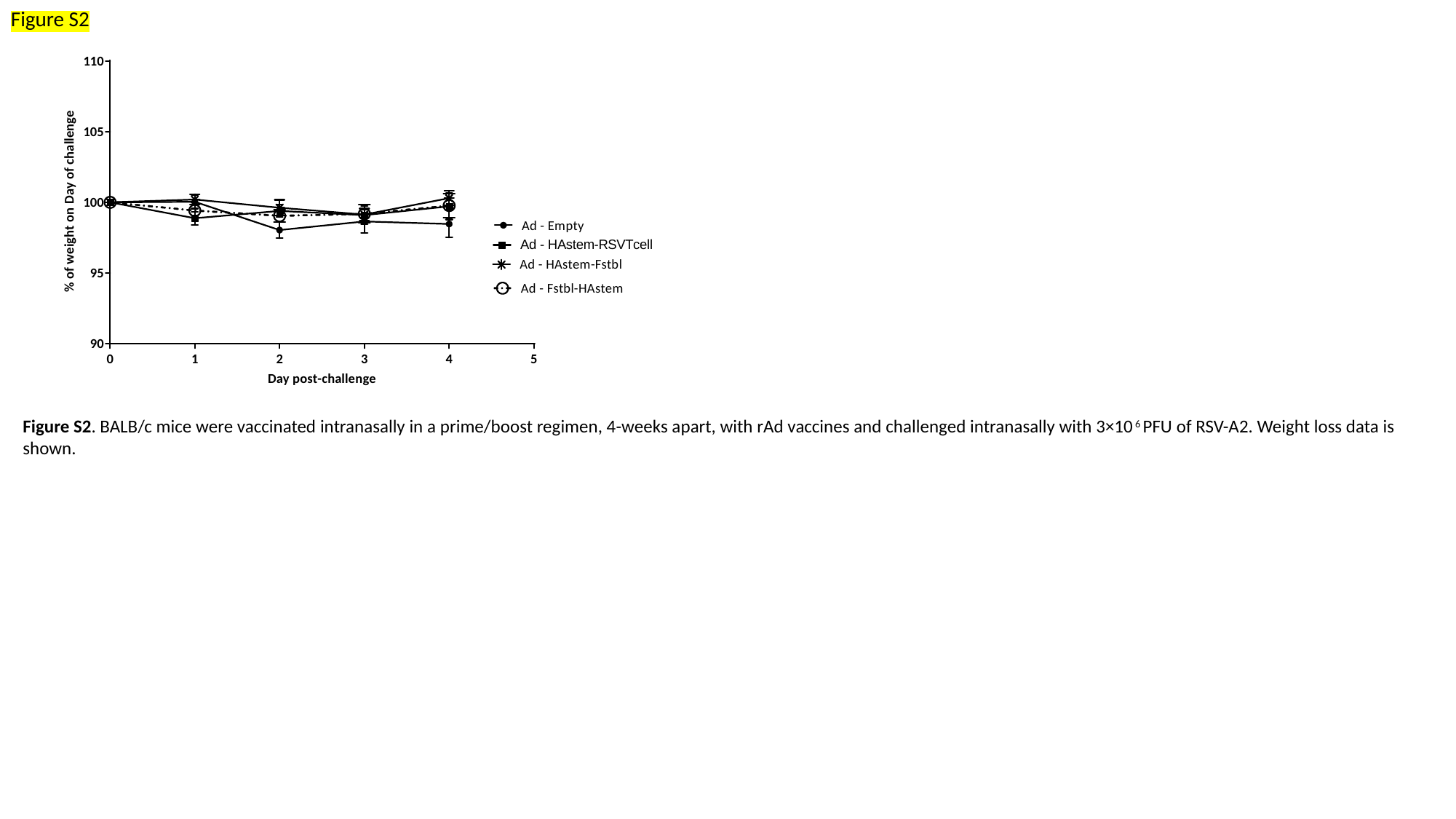

Figure S2
Figure S2. BALB/c mice were vaccinated intranasally in a prime/boost regimen, 4-weeks apart, with rAd vaccines and challenged intranasally with 3×10^6^ PFU of RSV-A2. Weight loss data is shown.
